# Vanishing Clams on an Iberian Beach: Local Consequences and Global Implications of Accelerating Loss of Shells to Tourism

**DOI:** 10.1371/journal.pone.0083615

**Published:** 2014-01-08

**Authors:** Michał Kowalewski, Rosa Domènech, Jordi Martinell

**Affiliations:** 1 Florida Museum of Natural History, University of Florida, Gainesville, Florida, United States of America; 2 Institut de Recerca de la Biodiversitat and Departament d'Estratigrafia, Paleontologia i Geociències Marines, Facultat de Geologia, Universitat de Barcelona, Barcelona, Spain; University of Western Sydney, Australia

## Abstract

Multi-decadal increase in shell removal by tourists, a process that may accelerate degradation of natural habitats, was quantified via two series of monthly surveys, conducted thirty years apart (1978–1981 and 2008–2010) in one small embayment on the Mediterranean coast of the Iberian Peninsula. Over the last three decades, the local tourist arrivals have increased almost three-fold (2.74), while the area has remained unaffected by urban encroachment and commercial fisheries. During the same time interval the abundance of mollusk shells along the shoreline decreased by a comparable factor (2.62) and was significantly and negatively correlated with tourist arrivals (r = −0.52). The strength of the correlation increased when data were restricted to months with high tourist arrivals (r = −0.72). In contrast, the maximum monthly wave energy (an indirect proxy for changes in rate of onshore shell transport) was not significantly correlated with shell abundance (r = 0.10). Similarly, rank dominance of common species, drilling predation intensity, and body size-frequency distribution patterns have all remained stable over recent decades. A four-fold increase in global tourist arrivals over the last 30 years may have induced a comparable worldwide acceleration in shell removal from marine shorelines, resulting in multiple, currently unquantifiable, habitat changes such as increased beach erosion, changes in calcium carbonate recycling, and declines in diversity and abundance of organisms, which are dependent on shell availability.

## Introduction

Local and global environmental impacts of tourism [1], [2], [3], [4] are intensifying due to rapidly expanding human populations [5]. Marine shorelines are particularly vulnerable because of the massive residential shift toward coastlines [6] and the fact that seashores remain among the most alluring tourist destinations [7], [8]. Tourists and single-day visitors *sensu* [9] affect beaches in diverse direct ways, including trampling, vehicle use, camping, shellfish harvesting, beach grooming, and many other factors [10], [11], [12]. However, long-term studies measuring human impacts on shoreline habitats are scant and fragmentary [10], [13]. In particular, the removal of dead shell remains by tourists represents one of the most understudied and least understood processes associated with human activities along marine shorelines.

Rigorous assessments of shell removal by tourists are needed because skeletal materials left behind by dead organisms perform many important environmental and ecosystem services. In pristine coastal habitats shells can occur in great abundance [14], [15] and serve multiple functions, from beach stabilization [16] to building materials for bird nests [17]. Also, shells may be intermittently submerged or transported into subtidal settings where they can provide various ecosystem services, including shelter for diverse endobiolithic algae [18], substrate for seagrass meadows [19], colonization sites for encrusting organisms [20], or armored protection for hermit crabs and fish [21], [22], [23]. Shell material is also continuously dissolved [24] in most coastal areas resulting in elemental recycling back into the global marine reservoir. The tourism-related removal of shell material from shorelines may thus have diverse environmental consequences.

Diverse processes can induce seasonal or decadal changes in shell abundance along shorelines and multiple hypotheses can be postulated *a priori* to explain variation in shell abundance through time. First, changes in local hydrodynamics may alter the magnitude of onshore transport of shell material [25], [26]. This model predicts a significant correlation between proxy measures of onshore transport (e.g., wave energy) and shell abundance. Second, changes in ecosystem structure and population dynamics (especially spawning and mortality patterns) may induce variation in local shell accumulation rates, which may be partly controlled by input of newly dead specimens [27]. This model predicts seasonal or decadal-scale correlations between temporal ecosystem changes and trends in shell abundance and also implies possible changes in rank abundance of dominant species or other ecological proxies (e.g., frequency of predation events and per-species size-frequency distributions). Finally, as postulated here, changes in tourism activities and tourism-related beach management practices may be responsible for changes in shell abundance. This last model predicts an inverse seasonal (or decadal) correlation between shell abundance and tourist arrivals.

In this study, local changes in shell abundance observed over the last 30 years were evaluated for one Mediterranean beach using two quantitative datasets from the same embayment, one collected more than 30 years ago and one collected in recent years. During a time interval of three years (7/1978 - 7/1981), quantitative surveys of shell material were conducted monthly along Llarga Beach Salou, Catalonia [28]. The same shoreline transects were resurveyed 30 years later (2008–2010). Because the sampled beach has not been altered considerably over the last 30 years, the resulting comparative data should be particularly suitable for testing the hypothesis that an increase in local tourism leads to the accelerated shell removal. Specifically, we evaluated the three models outlined above regarding changes in shell abundance in a local shoreline habitat. We tested for correlatives between the intensity of local tourism and shell abundance at two temporal scales: intra-annual/seasonal and multi-decadal. Within the same reference timeframe, we evaluated putative roles of other causative processes unrelated to tourism that could have led to shifts in shell abundance, either seasonally or through time, and explored global implications of the tourism-related removal of shells from coastal environments.

Our focus on removal of empty shells due to tourism in an area that has remained relatively unchanged differs in goals from studies targeting regions that experienced substantial ecosystem degradation through time [29]. Also, previous projects aimed at assessing the impact of human activities on live shellfish, including subsistence and recreational harvesting [30], [31], collecting for curio trade [32], bait collecting [33], or inadvertent trampling of live organisms [33]. Instead, we focused here on activities that may be intuitively perceived as least harmful: the impact of beachcombing (leisurely collecting, inadvertent trampling, use of recreational vehicles, etc.) on empty seashells scattered along marine shorelines.

## Materials and Methods

### Ethics Statement

The Llarga Beach is not included on the list of sites of natural interest protected by law and the endangered mollusk taxa (*Lithophaga lithophaga* (Linnaeus 1758) and *Pinna nobilis* Linnaeus 1758) have not been reported at the sampled locality. Consequently, the field study did not involve endangered or protected species. Live specimens were not collected in this study and permits were not required to collect modern shell material for scientific research in the study area [34]. Shell data used in this study have been archived as a PLoS One online-access appendix (Table S1).

### Study Area

**Figure 1:**
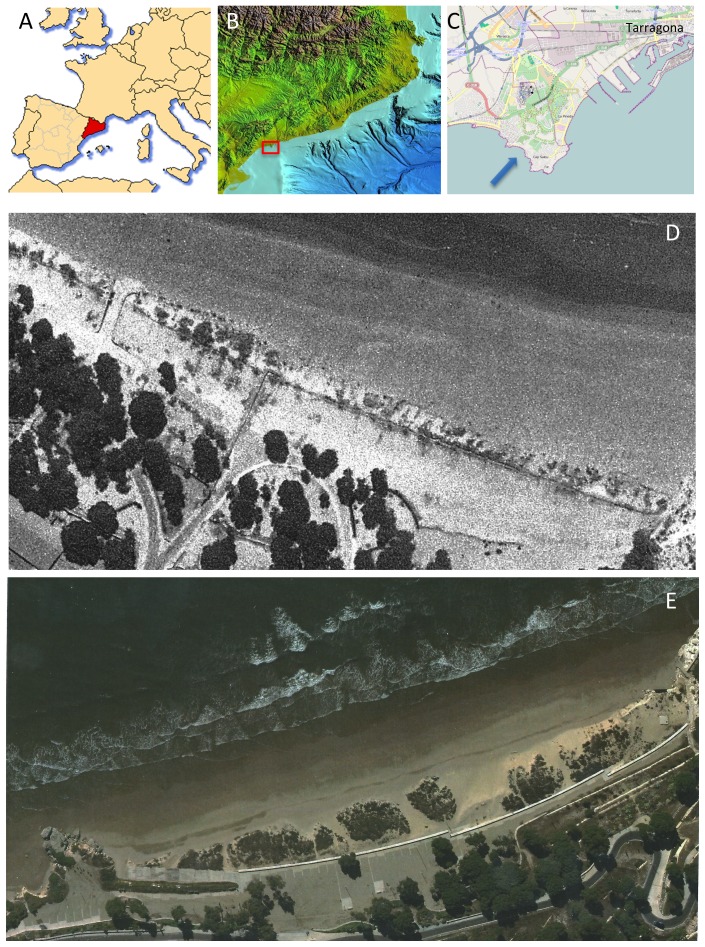
The study area. (a) A location map of the Catalonia Province; (b) A physiographic map of the Catalonia Province (non-copyrighted map, courtesy of Marine Geosciences Group, University of Barcelona); (c) A close-up map of the Llarga Beach (indicated with a blue arrow) and adjacent areas (image generated from an open access website: http://www.openstreetmap.org/); (d) an aerial photograph of Llarga Beach (flight # 10173, March 1, 1976; ©Cartographic Institute of Catalonia; reproduced with permission of the Cartographic Institute of Catalonia); (e) an aerial photograph of Llarga Beach (flight # 2011072000290012, February 18, 2011; ©Cartographic Institute of Catalonia; reproduced with permission of the Cartographic Institute of Catalonia).

Llarga Beach (*Platja Llarga*) is a sandy beach ∼600 m in length and ∼25 m in width. It is bounded by a Jurassic limestone to the west and delineated by Pleistocene eolian sandstone outcrops in the central and eastern part of the beach (Fig. 1). None of those rock units contribute shells that could be confused with modern shell material. The fossil record of the Jurassic limestone in the area is represented by scarce belemnite guards, ammonites molds, and fragments of oyster shells. The Pleistocene sandstone contains small gastropods and bivalves readily distinguishable from modern materials based both on taxonomic and taphonomic (i.e., alterations due to fossilization processes) criteria [28].

The satellite images indicated that beach topography, its aerial extent, and vegetation have remained virtually unchanged (Fig. 1) over the 30 years that separated the two intervals of monthly surveys reported here. Also, the embayment has not been altered by direct urban encroachment, commercial activities, fisheries, and shellfish harvesting, which all concentrate in other areas of the Mediterranean coast of Spain. Shellfish fishery that could affect locally common bivalves such as *Chamelea gallina* (Linnaeus, 1758) and *Donax trunculus* (Linnaeus, 1758) has been intermittent and mostly concentrated in other areas located much farther south (90 km) or much further north (260 km) from the study area (Castelló, F., 2012, pers. comm).

**Figure 2:**
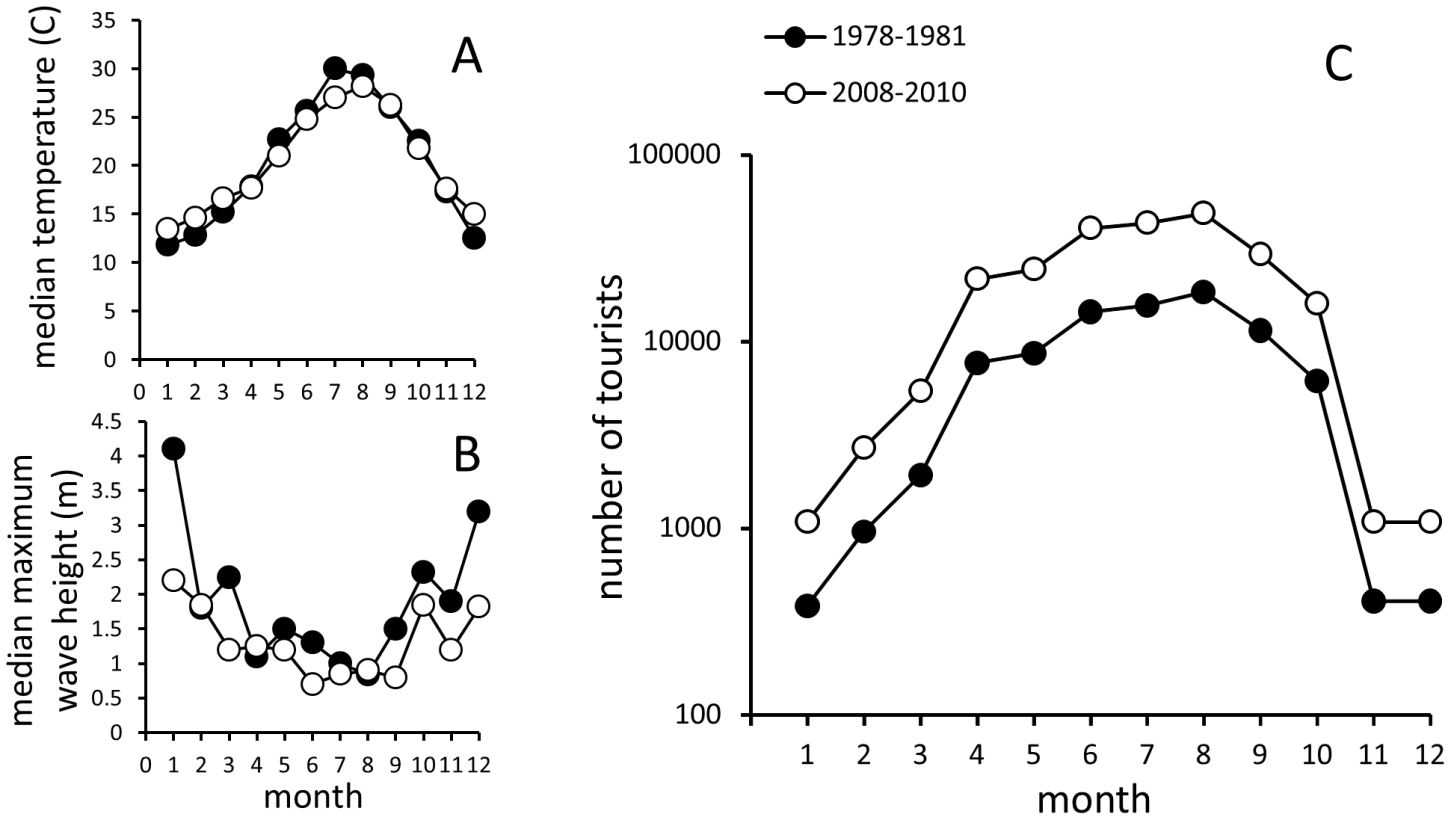
Local temperature, wave height and tourist arrival data for the studied time intervals. (a) Median monthly air temperature recorded at the Meteorological Station of Reus Airport (data provided by Servei Meteoròlogic de Catalunya); (b) Median maximum monthly wave height measured at a buoy (Simar-44 [2057048]) located proximally to the study area (publicly available data obtained from http://www.puertos.es; Puertos del Estado, Ministerio de Fomento); (c) A log-linear plot of monthly tourist arrivals for the 1978–1981 and 2008–2010 time intervals (see text for data sources).

Llarga Beach has not changed notably in terms of local weather and hydrodynamics (Fig. 2): neither average monthly temperature (Fig. 2A) nor wave height (Fig. 2B) changed substantially over those 30 years. In contrast, the area has seen a nearly three-fold increase in tourist arrivals across all months (see below for data sources). A median number of local tourist arrivals increased by a factor of 2.74 over the last 30 years, while relative monthly changes in tourist arrivals have remained virtually identical (Fig. 2C).

In summary, over the last three decades, a small coastal area represented by Llarga Beach has experienced a considerable increase in tourist arrivals, but has remained relatively static otherwise and should provide a useful model system for assessing the localized impact of tourists on abundance of shells in coastal environments.

### Shell assemblages

Primary data were derived by systematic transect sampling of mollusk material present on the surface of shoreface zone. Qualitative visual surveys of faunal composition were performed continuously throughout the field work. These qualitative assessments indicated consistently that three species of bivalves (*Chamelea gallina, Donax trunculus,* and *Donax semistriatus* Poli, 1795) dominated local beach assemblages throughout the sampled time interval. Consequently, their combined abundance should represent a robust proxy for abundance of all shell material along the shoreline. Only those three species have been targeted in quantitative analyses reported here. All other species observed during sampling were present intermittently, and only three were present occasionally in notable quantities: *Spisula subtruncata* (da Costa, 1778), *Mactra stultorum* (Linnaeus, 1758), and *Acanthocardia tuberculata* (Linnaeus, 1758). Other bivalve species observed occasionally included *Glycymeris insubrica* (Brocchi, 1714), *Chlamys varia* (Linnaeus, 1758), *Tellina planata* Linnaeus, 1758, *Scrobicularia plana* (Da Costa, 1778) and *Ensis ensis* (Linnaeus, 1758).

The three dominant species included *C. gallina*, which is restricted to the Mediterranean and Black Seas, *D. semistriatus*, which also appears on Atlantic coasts of the Iberian peninsula, and *D. trunculus*, which is known from littoral habitats from the Great Britain to Senegal. In the Mediterranean, all three species occur in shoreface sand communities (SFC *sensu* [35]). Both donacid species occur together exclusively in this habitat, whereas *C. gallina* is also present in subtidal sands down to 20 m depth (SFBC community *sensu* [35]). *C. gallina* is a mobile suspension feeder and the reproductive cycle of this commercially important species has been studied extensively in the Mediterranean [36], [37]. *C. gallina* appears to reproduce primarily in the summer (or both spring and summer), with one or two peaks of gamete emission [36]. *D. trunculus* and *D. semistriatus* are detritivorous species. *D. trunculus*, the larger and more abundant of the two species, spawns from March-April to October in unpolluted areas of the southern Mediterranean littoral [38], [39].

Both *C. gallina* and *D. trunculus* are a food source for carnivorous gastropods, especially naticid gastropods, as demonstrated by the ubiquitous presence of valves with drillholes characterized by a countersunk shelf (*Oichnus paraboloides* Bromley, 1981). Shells of the common drilling predator (*Natica hebraea* Martyn, (1784)) have been collected sporadically on Llarga Beach.

### Sampling Methods

Monthly surveys were conducted during two time intervals: (1) 1978–1981 and (2) 2008–2010. During the first time interval the surveys were conducted every month from July 1978 through July 1981 (37 monthly transects total). Due to time and funding constraints, surveys conducted during the second time interval were more limited and included transects conducted monthly from August 2008 through October 2008 and from July 2009 through June 2010 (15 monthly transects total). For both time intervals, every calendar month was represented by at least one transect. Samples collected during those surveys were retained and are currently housed at the University of Barcelona.

In both time intervals, monthly surveys were carried early in the morning on weekdays near the end of each month along the 300×10 m (3000 m^2^) shoreline transect with its endpoints defined by a carbonate cliff at the west end of the beach and a sandstone outcrop located centrally (Fig. 1). All transects were conducted along a 10 m wide swath most proximal to the waterline. Because of microtidal regime, daily tidal oscillations in the region do not exceed 10 cm and show a minimal annual variation [40]. Thus, tidal variation resulted in minimal lateral shifts from one survey to another, when using water line as a reference system.

Quantitative surveys focused on the three dominant species used as a proxy for the overall quantity of coarse biogenic material present on the beach (see above). The surveys were carried out by exhaustive sampling of all specimens found on the surface within the transect area. No subsurface material was counted or collected. Species identity and evidence of predation (i.e., presence/absence of drill holes) were recorded for each specimen. In addition, shell length was measured using digital calipers for specimens of *C. gallina* for each August transect available for each time interval. The resulting variables (relative abundance of dominant species, drilling frequency, and body size-frequency distributions) provided proxies of ecological processes for local benthic communities. This approach is justified here by growing evidence that shell assemblages can approximate local benthic ecosystems with high fidelity [41], [42]. Even intertidal shell assemblages, including beach materials, can effectively capture information on local biodiversity [43], [44].

Tourist arrivals were estimated using data available from town hall publications and unpublished counts (S. Antón 2013, University of Barcelona, pers. comm.) for lodging facilities directly adjacent to Llarga Beach. The arrival estimates were compiled to assess seasonal changes in tourism, including monthly estimates, seasonal estimates (“high season” represented by summer months and “low season” represented by non-summer months), and annual estimates. All local facilities existed throughout both of the studied time intervals. The only exception was the campground, which was active in 1978–1981 time interval but closed permanently before the second sampling time interval. The campground has been replaced by a private recreational area with a low density of occupation. The number of arrivals is likely an underestimate of the actual number of tourists visiting Llarga Beach because same-day visitors were not accounted for by the metric used here. However, estimates of tourist arrivals were used here only as a relative metric of changes in intensity of tourism across seasons and through time.

### Analytical Methods

To test the three models postulated above, repeated sampling surveys of the same transect were conducted monthly across seasons for two time intervals ∼30 years apart. This approach generated a series of shell abundance estimates that can be assessed for correlatives against parallel datasets representing potential controlling factors, including corresponding trends in tourist activities, local physical characteristics, and indirect ecological proxies. Whereas additional spatial control for the same time interval (ideally derived from comparable habitats inaccessible to tourists) would have strengthened the research design, such data were not available. Consequently, all causal interpretations proposed below are tentative and should be viewed as initial hypotheses that require further testing in other settings.

The data (Table S1) resulting from the repeated surveys were analyzed by time interval and seasonally within each time interval. Seasonal changes were estimated by averaging monthly or bimonthly data within each time interval (e.g., a bimonthly January-February estimate for the 2008–2010 time interval was computed as an arithmetic mean based on all available 2008–2010 January and February transects). Data were also grouped into non-tourist (September-May) and tourist (June-August) months. Bimonthly averages were used in some analyses to increase sample size because, for certain months, only one transect was available in a given time-interval (e.g., only one February transect for 2008–2010 time interval). The resulting time series were analyzed using raw values. In addition, first differencing was applied in order to detrend time series.

Non-parametric rank-based methods (Spearman rank correlation) and permutation tests were used as a primary tool in statistical testing. These methods were selected because some of the analyzed variables (e.g., wave energy, tourist arrivals) represented notably skewed distributions, sample sizes were small in some cases, and groups varied in sample size. The parametric approach (2-Way Unbalanced ANOVA) was applied in one case to evaluate differences in mean shell size for monthly and decadal data groups. This approach was deemed appropriate because groups had comparable dispersions (standard deviation ranged from 2.45 to 5.07 mm), all distributions were unimodal and did not display pronounced departures from normality (absolute values of skewness and kurtosis were <2 in all cases), and sample sizes were relatively large (see Results section below) further minimizing the effect of slight non-normality that may have affected the data. However, because of notable variation in sample size across groups, an unbalanced ANOVA was employed (GLM procedure, SAS).

A significance level of alpha = 0.05 was used for hypothesis testing and Bonferroni correction for multiple tests was applied when appropriate. As used here, significance values (p) denote probability of Type I Error and should not be misconstrued as indicating likelihood estimates in support of a given null hypotheses. SAS software, PAST freeware, and SAS/IML programming language were used in all statistical analyses.

## Results

**Figure 3:**
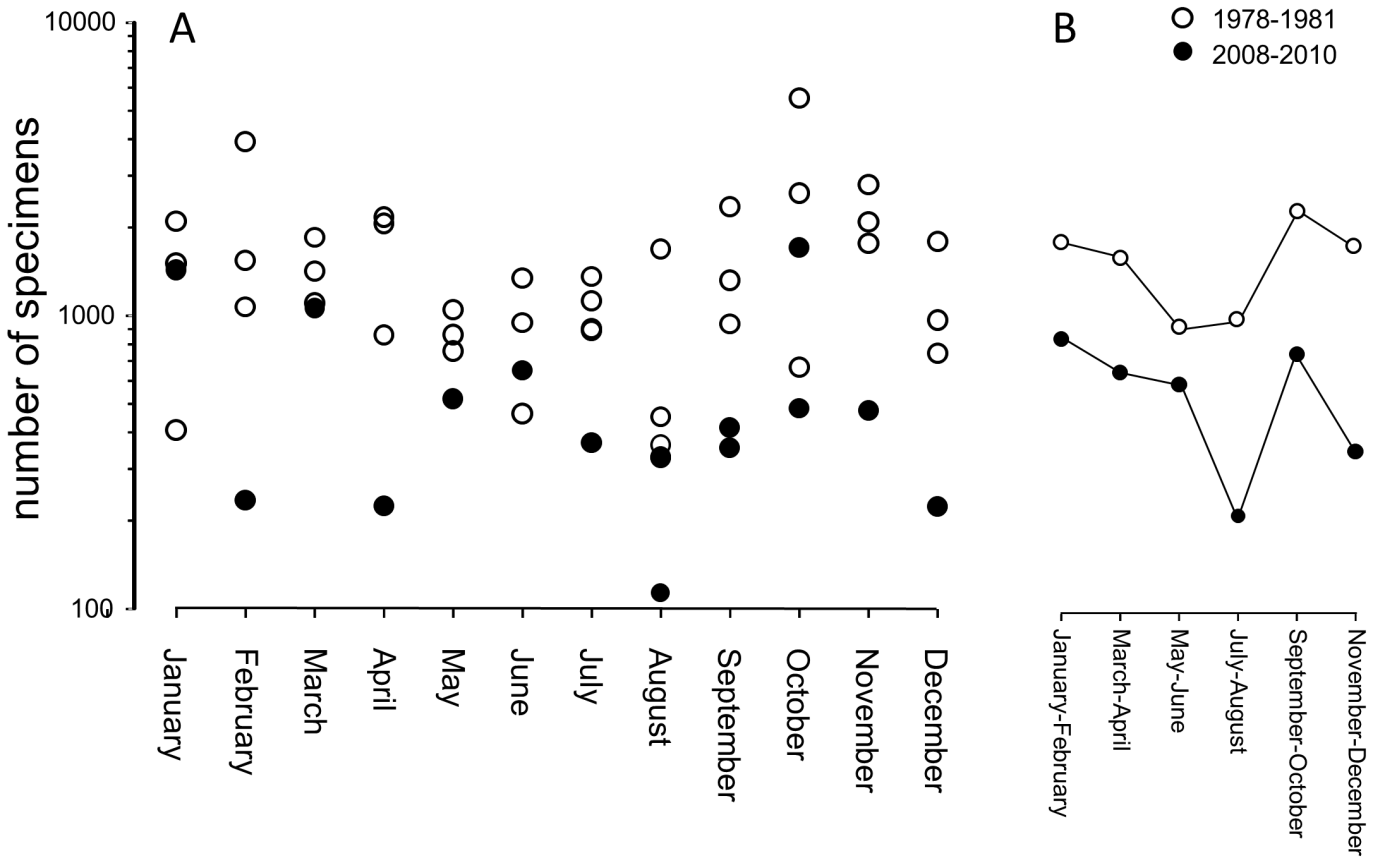
Seasonal changes in abundance of common bivalve species based on transects conducted along the shoreline of the Llarga Beach. (a) A bivariate log-linear plot of monthly changes in abundance of common bivalve species based on transects conducted along the shoreline of the Llarga Beach during 1978–1981 and 2008–2010 sampling intervals. Each point represents a single monthly transect; (b) A comparison of bimonthly changes in average abundance for 1978–1981 versus 2008–2010 sampling intervals.

A comparison of 1978–1981 and 2008–2010 surveys indicated that shells at the shoreline of Llarga Beach (Fig. 3) were almost three times more abundant three decades ago: on average, 1506.5 specimens were recovered per transect in 1978–1981 compared to only 578.3 specimens in 2008–2010. The decline in shell abundance was comparable in magnitude for the tourist season (July–August) (70.1%) and for other months (60.0%). When 1978–1981 and 2008–2010 transect estimates were binned bimonthly (due to small number of transects for some of the winter months, monthly bins were inadequate), there was a remarkable congruence in seasonal changes in shell abundance with notable declines in shell quantity observed in the late spring, summer, and the late fall (Fig. 3B). One notable difference between the two time-intervals was a much steeper decline in average specimen abundance, which was observed only for the July-August months for the 2008–2010 sampling interval (Fig. 3B).

The maximum monthly wave energy [MMWE] for 1978–1981 and 2008–2010 was not correlated significantly with shell abundance [SA] (Spearman *r* = 0.10, *p* = 0.46) and the pattern persisted for first differences (month-to-month changes) [Δ_SA_] (*r* = −0.18, *p* = 0.21). When MMWE values were downweighted by the length of a time lag between the last day in which wave height was at monthly maximum and the day of sampling [MMWE* = MMWE/#days], correlations remained non-significant: (SA: *r* = 0.26, *p* = 0.06 and Δ_SA_: *r* = −0.23, *p* = 0.10, respectively). All correlation coefficients were low (*r*<0.3), the first differences [Δ_SA_] yielded correlation coefficients with an opposite sign relative to the raw correlations [SA], and none of the tests was significant. When data were subdivided by season, time-interval, or both, correlation coefficients either remained low or were non-interpretable due to excessively small sample sizes.

**Figure 4:**
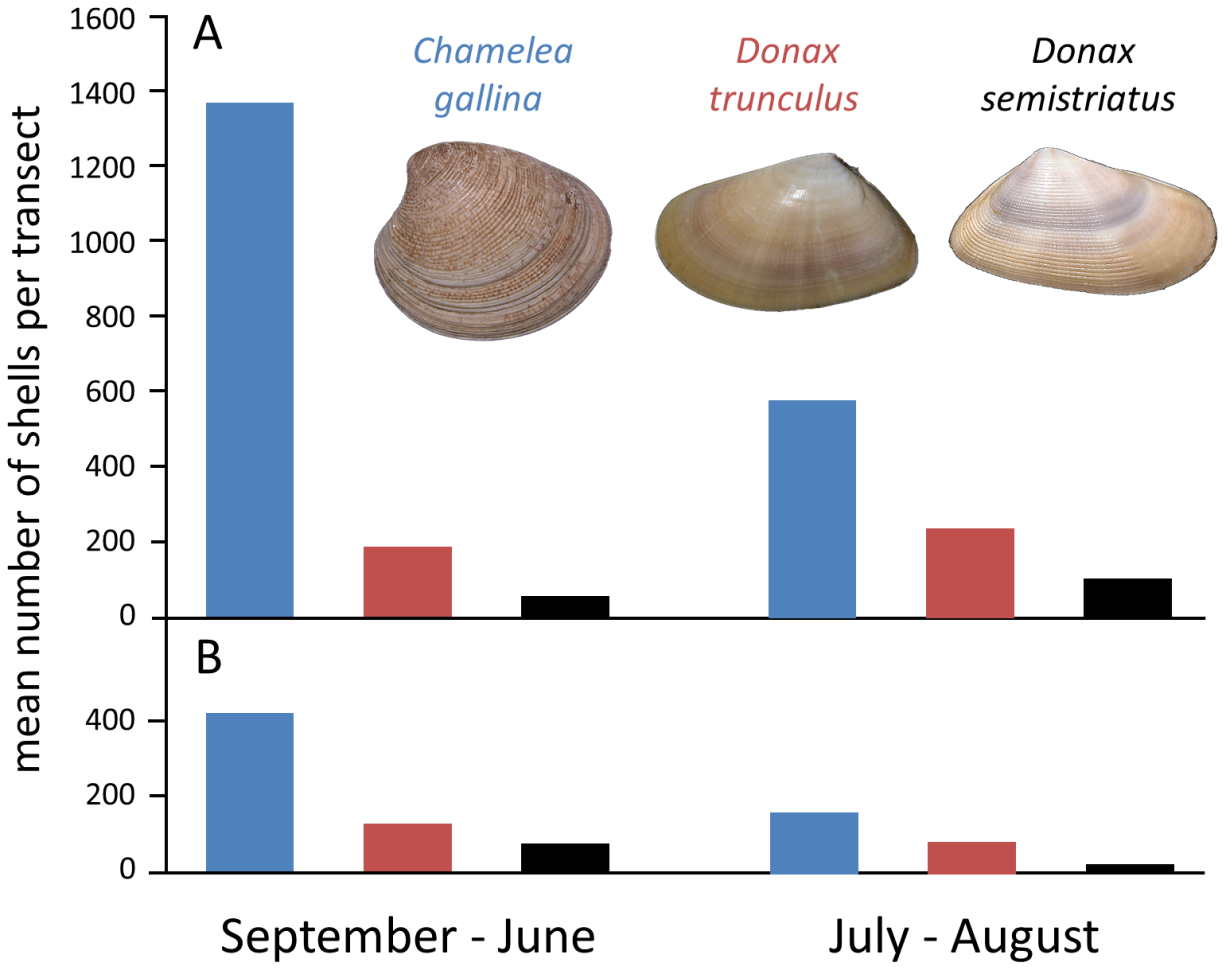
Rank abundance of shells of the three dominant species grouped by tourist seasons for the 1978–1981 and 2008–2010 sampling intervals. Values averaged across all transects conducted during a given time interval and season. (a) Data for 1978–1981 transects; (b) Data for 2008–2010 transects.

**Figure 5:**
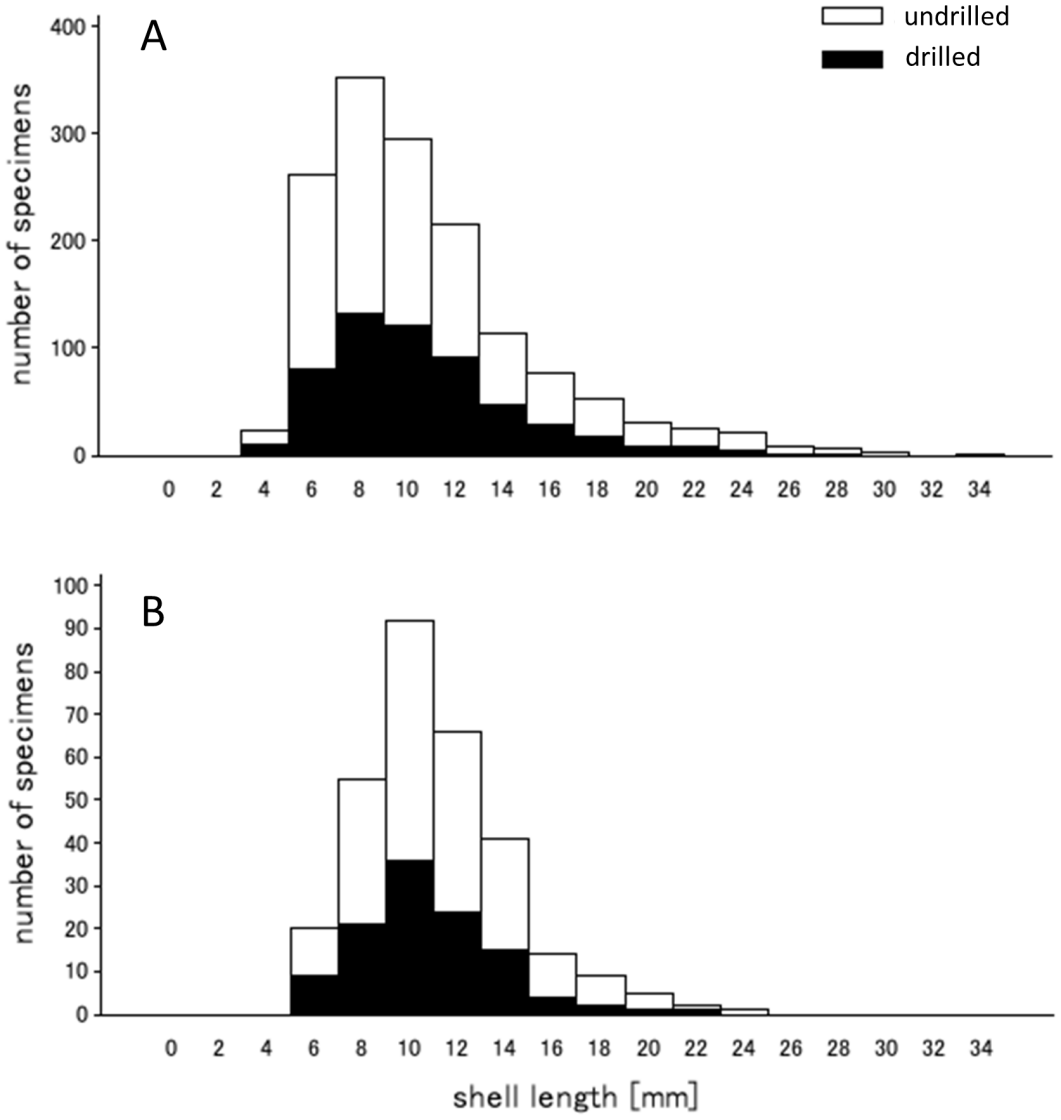
Size-frequency distributions of shells of *Chamelea gallina* from August transects. Drilled and undrilled specimens are differentiated. (a) Data for 1978–1981 time interval; (b) Data for 2008–2010 time interval.

No dramatic changes in indirect ecological proxies measurable from shell assemblages could be demonstrated across seasons or through time. Whereas the absolute abundance of specimens per transect decreased notably over the last 30 years (Fig. 4), the rank abundance of the three dominant species remained unchanged regardless of the season, with *C. gallina* being the dominant species and *D. semistriatus* the least abundant species (Fig. 4). Similarly, drilling frequency, a proxy for predator-prey interactions between carnivorous gastropods and bivalves, showed consistent patterns through time. *C. gallina* was drilled frequently in both time intervals (48.3% of drilled valves in 1978–1981 and 41.7% of drilled valves in 2008–2010, respectively), *D. semistriatus* was drilled infrequently (8.2% for 1978–1981 and 8.9% for 2008–2010), and *D. trunculus* was drilled rarely (1.3% for 1978–1981 and 4.3% for 2008–2010). Seasonal changes in drilling frequency also persisted through time. For both time intervals and for each species, drilling frequencies were notably lower during summer months. Finally, comparison of size frequency distributions for specimens of *C. gallina* obtained for August transects did not suggest any dramatic shifts in body size between the two time intervals (Fig. 5). The monthly median values for valve length varied in a comparable range for 1978–1980 August transects (*n* = 826, M_1978_ = 8.11, *n* = 205, M_1979_ = 12.48, *n* = 452, M_1980_ = 12.30) and 2008–2010 transects (*n* = 259, M_2008_ = 10.69, *n* = 46, M_2008_ = 9.99). Moreover, the variation in median shell size was more variable between the transects within the 1978–1980 time interval than across the two time intervals. The maximum difference in medians for 1978–1980 was 3.19 mm (M_1980 vs._ M_1979_), whereas the maximum difference in medians observed between the two time intervals was 2.58 mm (M_1978 vs._ M_2008_). When data were grouped by both, time interval and month, the two time intervals did not differ significantly from one another, once among-transect variation within time intervals was considered explicitly (Unbalanced 2-Way Anova, df = 1, MS = 40.27, F = 2.85, p = 0.09).

**Figure 6:**
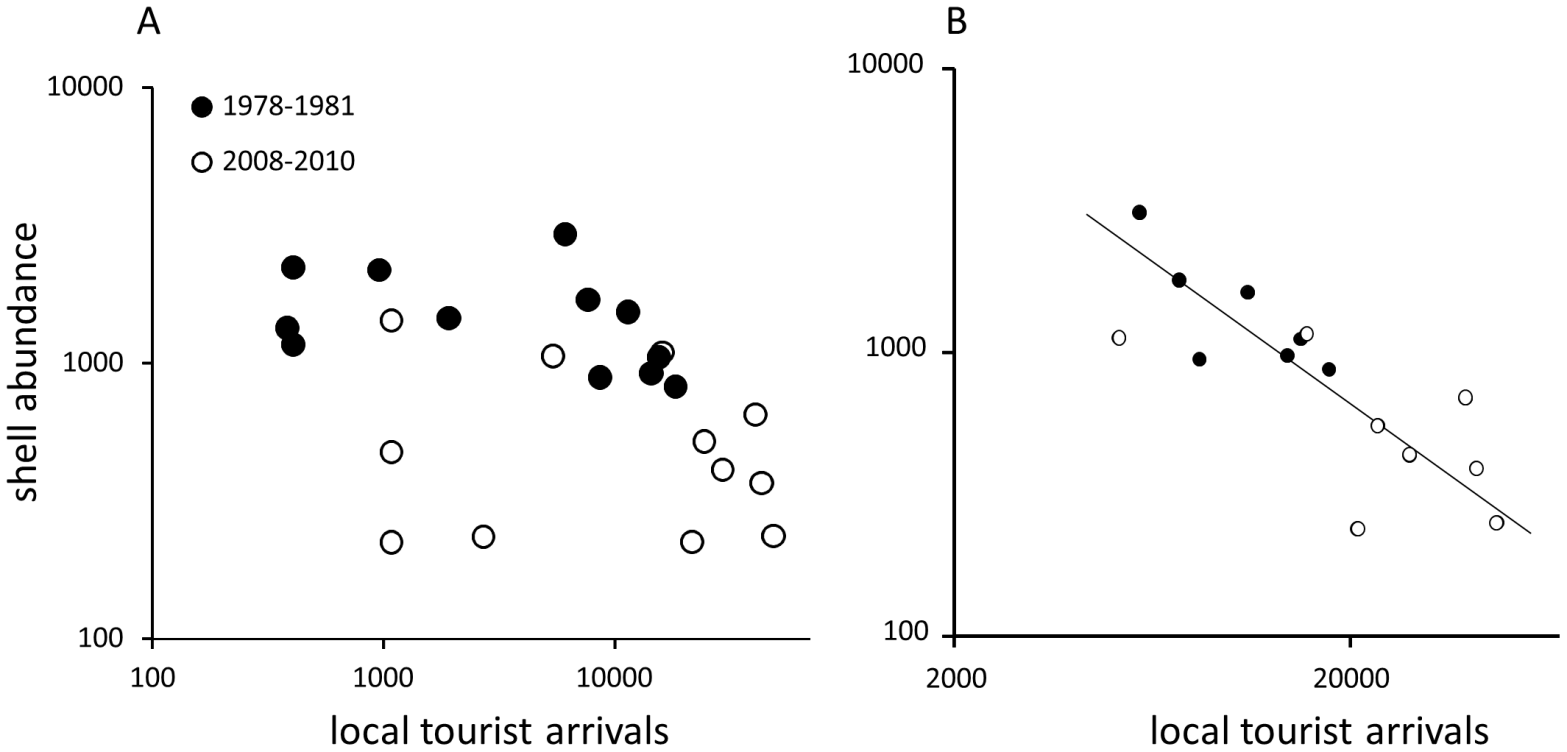
Log-log bivariate plots of tourist arrivals and shell abundance. Each data point represent average for the same months sampled in a given time interval (e.g., all July transects obtained in the 1978–1981 sampling period will be represented by one mean value. (a) All data; (b) Data restricted to months with tourist activity >5000 (a solid line represents a Reduced Major Axis Regression model).

When shell abundance estimates for each transect was compared with corresponding local tourist arrivals, significant negative correlations were observed for monthly data (n = 51, r = −0.52, p<0.0001) and for data averaged by month within each time interval (n = 24, r = −0.49, p = 0.015). Absolute values of correlation coefficients were relatively modest and the bivariate interrelation was not strong when examined visually for data averaged by month (Fig. 6A). However, the tourist arrivals were distinctly bimodal (note a gap along x-axis of Fig. 6A). When data were restricted to months when tourist arrivals had been high (>5000), a strong negative correlation was observed (Fig. 6B), whether analyzed for monthly data (n = 32, r = −0.72, p<0.0001) or averaged by month within each time interval (n = 15, r = −0.84, p = 0.0001). For restricted data, tourist arrivals were a reasonable predictor of shell abundance (r^2^ = 0.64, Reduced Major Axis Regression, p = 0.0008, Permutation Test). When data were analyzed separately for each time interval, correlation coefficients remained negative, although not always significant (as may be expected given a loss of statistical power associated with reduced sample size). Correlations between first differences (month-to-month change in shell abundance versus month-to-month change in tourist arrivals) were also negative, but less pronounced and non-significant in most cases. Because adjacent transect estimates were separated on average by 30 days, they did not represent directly adjacent time series datapoints with high potential for strong co-dependences. Consequently, detrending may be an overly conservative and potentially misleading corrective strategy.

**Figure 7:**
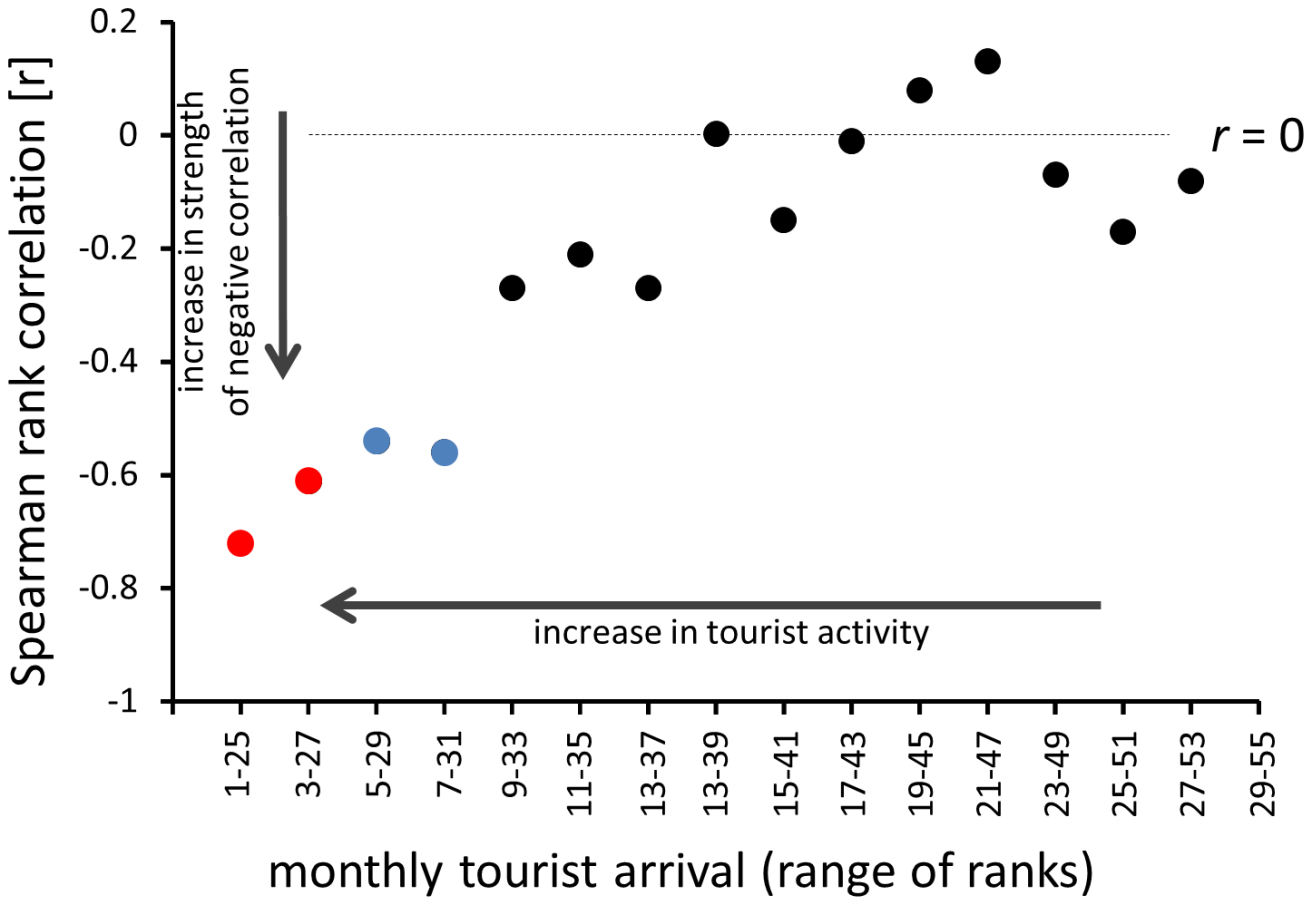
Changes in strength of rank correlation as a function of tourist activity. Monthly estimates are binned into 25-transect groups based on their rank in terms of tourist arrivals. The left endpoint along the x axis (ranks “1–25”) represents the dataset restricted to 25 monthly transects with the highest tourist arrivals. The right endpoint (ranks “29–55”) represent the 25 months with the smallest numbers of tourists. All restricted datasets are comparable in sample size (*n* = ∼25 transects) although due to tied ranks, some rank ranges represent 24 or 26 monthly transects. Red symbols are significant at *p*<0.001 and blue symbols at *p*<0.01.

When data were grouped by tourist arrivals, the strength of correlation increased as a function of tourist arrivals (Fig. 7). The Spearman correlation coefficient reached the most negative value for data restricted to 28 months with the highest tourist arrivals and fluctuated around 0 for months with the lowest tourist arrivals (Fig. 7).

## Discussion

### Causes of Decline in Shell Abundance

The three models of shell decline evaluated in this study include trends in hydrodynamics resulting in changes in rates of onshore transport of shell material; ecological shifts in population dynamics and ecosystem characteristics resulting in time-variant shell input; and changes in tourism activities and tourism-related beach management practices resulting in varying rates of shell removal from shoreline areas. Whereas these models do not represent an exhaustive list of causal explanations, many other obvious factors such as changes in beach geomorphology, urban encroachment, or an increase in commercial fisheries are unlikely to apply. Other possible causal drivers are expected to be reflected in patterns evaluated by the three postulated models (e.g., an invasive species could alter rank order of dominant species, thus supporting ecological changes through time).

Seasonal changes in wave energy may result in differential delivery of shell material to the beach [25], [45] and decadal-scale changes may consequently produce similar changes over longer time scales. Because wave energy has remained relatively unchanged over the last 30 years and shell abundance did not show any significant correlations with wave height regardless of data groupings and tests, it is unlikely that changes in local hydrodynamic played a notable role in the observed decline in shell abundance.

Population dynamics of the three studied species may have varied seasonally or through time, resulting in a variable input of new shell material to the beach. Because the initial shell input into death assemblages is expected to be primarily controlled by biological productivity [46], [47], including spawning patterns, mortality rates, and related locally-controlled processes, seasonal and multi-decadal changes in shell input at Llarga Beach cannot be evaluated directly (bivalve population data for the study area were not available). However, over the last 30 years the same three species have dominated the local shell assemblages and have shown comparable seasonal trends. It is, thus, unlikely that the observed decline in shell abundance in the last three decades was driven by changes in population dynamics. Moreover, for each of the three species size frequency distributions remained comparable through time, suggesting temporal stability in mortality and recruitment patterns. Finally, patterns of drilling attacks by predatory snails (within and across prey species) did not change notably between the two time intervals. Thus, all indirect ecological proxies measurable from the sampled shell assemblages consistently suggested that no major changes took place in local ecosystems over the last 30 years.

A significant negative correlation was found between tourism and shell abundance consistently at several levels: (1) over the last 30 years, shell abundance declined almost three-fold (2.62), a value which is remarkably close to the concurrent increase in local tourist arrivals (2.74); (2) seasonally, the substantial increase in summer tourism was congruent with concurrent decline in shell abundance; and (3) monthly, negative correlation between shells and tourist arrivals was observed, especially for months for which local tourist arrivals are high. The increase in strength of the correlation with increase in tourist arrivals is particularly compelling because it suggests that when tourist activity was high shell abundance decreased. Conversely, when local tourist arrivals were low, the tourism-associated shell loss was less important than other processes that may have contributed to variation in rates of shell accumulation and shell removal.

### Active collecting versus other shell removal processes

The tourism-related processes that contribute to shell removal are certainly not limited to shell collecting, although collecting is an important activity along marine shorelines [48], as also demonstrated by hundreds of published shell guides aimed at leisurely and avocational shell collectors. However, other processes may be also important, including trampling [10], [33], recreational clam harvesting [49], use of recreational vehicles [12], sand dredging for beach recovery [10], and other similar activities. In addition, beach maintenance, which can be expected to correlate with tourism intensity, often involves extensive cleaning and grooming of beach areas with heavy equipment [10], [29], [50], [51]. This is the case for Llarga Beach, where maintenance activities, using tractors with rakes, occurred daily during summer months of 2008–2010, but not during the 1978–1981 sampling interval. These maintenance activities may also explain the anomalously high decline observed for the 2008–2010 July-August months when comparing with the 1978–1981 time interval (Fig. 3B).

The relative importance of these various tourism-related processes is impossible to evaluate for Llarga Beach due to lack of relevant data. In fact, we are not aware of any published assessments addressing this issue for dead shell material (there exist numerous studies on subtidal harvesting of live mollusks for curio trading [32], [52] or impact of other tourism-related processes on beach and intertidal communities [10], [11], [12], [50], [51]). The results reported here suggest that an increase in tourism can trigger a major decrease in shell abundance along shorelines thus providing justification for future studies exploring specific processes that contribute to removal of shells from beaches by tourists.

As mentioned above, other human-related activities such as commercial fisheries are unlikely to have played a significant role in the study area. Although *C. gallina* and *D. trunculus* are commercial species, fisheries along this coast have been operating over the last three decades in areas located far away from Llarga Beach (Castelló, F., 2012, pers. comm).

### Local Shell Removal as a Proxy for Global Trends

Over the last 30 years, global tourism has grown four-fold, from ∼250 million tourist arrivals in 1980 to ∼billion arrivals in 2010 (UNWTO Tourism Highlights 2010 by the World Tourism Organization). The nearly three-fold increase in tourist arrivals at Llarga Beach is thus not unusual. Given this increase in intensity and diversity of tourism-related processes in coastal habitats [10], [11], [12], it is likely that shell abundance has decreased on many marine shorelines, paralleling shell losses due to curio trading [52], [53]. In fact, some shell-rich countries that experience intense tourism have already recognized the severity of this problem and explicitly regulate not only the type, but also the quantity, of shell material that visitors are allowed to export out of the country (e.g., the Commonwealth of Bahamas). However, we lack assessments of impact of tourism on shelly organisms over multi-decadal time scales [10] largely because of the absence of methodologically comparative samples from past decades [29]. Estimates reported here may serve as a starting point toward developing empirical estimates for shell loss due to tourism.

It is likely that the nearly three-fold decline in shell abundance observed at Llarga beach is a conservative estimate compared to other beaches visited by tourists. While a popular tourist area, Llarga Beach is not a highest-tier tourist destination and, as noted above, the increase in tourist arrivals observed for Llarga Beach was well below global estimates for the same time interval. Llarga Beach provides shell material dominated by small, common bivalves that are neither attractive to professional shell collectors nor spectacular enough to attract attention of every casual beachcomber. Compared to other beaches, which may harbor esthetically appealing specimens, Llarga Beach is unlikely to trigger enthusiastic collecting by tourists or motivate intense subtidal harvesting of live fauna for curio trading. Finally, the negative effects of collecting live specimens for subsistence and recreational shellfish harvesting [10], [31], [54] have been minimal in the study area compared to many other marine shorelines.

### Eco-Environmental Consequences

Removal of shell material from shorelines may trigger negative eco-environmental changes. Among others, such changes may include physical, chemical, and biological alterations.

For example, shell material scattered along the shorelines may stabilize sediments by forming pavements that hamper sediment movement [55], although eolian transport of fine fraction may be enhanced locally around coarse objects such as shells [56]. Selective removal of macroscopic shells may change the rates and patterns of sediment erosion along the shoreline.

Similarly, removal of shells potentially changes calcium carbonate budget. The carbonate skeletons of marine macro-organisms such as echinoderms and mollusks tend to be overlooked in modeling the global CaCO_3_ cycles in the oceans [57], although their role may be significant [58], [59]. The importance of mollusks in carbon cycle has been also acknowledged by listing them as a potentially important target for carbon sequestration [59]. As our understanding of the role of mollusks in the global elemental cycling continues to improve, we will also need to account for the removal of large quantities of mollusk shells from the natural cycle by tourism. If the removal of shells due to tourism along shorelines increased three-fold over the last three decades, as this study potentially implies, the tourists may be significantly altering the CaCO_3_ cycling on our planet.

Removal or destruction of shells, which serve variety of ecosystem functions, may also negatively affect a broad spectrum of organisms. The obvious examples include use of shells for anchoring by seagrass [19], settling for diverse encrusting organisms [20], [60], [61], and dwelling and mining by endobiolithic algae [18] and bioeroding sponges [62], [63]. Hermit crabs and fish also rely heavily on mollusk shells (mostly gastropods) and are often limited by lack of suitable shells [21], [22], [23]. Shells also serve as building materials for variety of coastal organisms, both terrestrial [17] and marine [64], and perform many other ecosystem services to a diverse array of organisms. It is fair to note here that the increase in shell abundance can also have negative effects. For example, beetles of the genus *Bledius* do not live in sand too rich in shells because of their digging behavior [65], [66] and sediments with a high fraction of shell fragments may negatively affect intertidal clams [66], [67].

## Conclusions

By exploiting a unique opportunity for developing comparative data on shell abundance for one specific coastal area on the Iberian Peninsula, the study demonstrated that shell abundance declined substantially over the last three decades. Moreover, the shell abundance patterns showed strong negative correlations (over multiple time scales) with tourist arrivals suggesting that tourism and tourism-related activities may be a driving force behind the accelerating removal of shells from marine shorelines. The shell loss may have substantial ecological and environmental consequences, although a rigorous quantitative assessment is not yet possible.

There is a growing realization that the most recent fossil record, including surficial skeletal remains found on land and in the sea, may yield diverse data for assessing ecosystem changes [14], [15], [41], [68], [69]. This study suggests that shell accumulations found along shorelines may also provide quantifiable information about local consequences and global implications of seasonal and multi-decadal changes in tourism.

## Supporting Information

Table S1 **Raw data on shell abundance used in this study.** Abbreviations: RD - number of right drilled valves; LD- number of left drilled valves; RUD - number of right non-drilled valves; LUD - number of left non-drilled valves; D - total number of drilled valves; UD - total number of non-drilled valves; R - total number of right valves; L - total number of left valves; Total - Total number of valves.
(XLSX)
